# Misclassification of Plasmodium infections by conventional microscopy and the impact of remedial training on the proficiency of laboratory technicians in species identification

**DOI:** 10.1186/1475-2875-12-113

**Published:** 2013-03-27

**Authors:** Peter Obare, Bernhards Ogutu, Mohammed Adams, James Sande Odera, Ken Lilley, David Dosoo, Christine Adhiambo, Seth Owusu-Agyei, Fred Binka, Elizabeth Wanja, Jacob Johnson

**Affiliations:** 1 Kenya Medical Research Institute/United States Army Medical Research Unit, Kenya, Malaria Diagnostics Centre, Box 54 - 40100, Kisumu, Kenya; 2 Kintampo Health Research Center, Box 200, Kintampo, Brong Ahafo Region, Ghana; 3 Australian Army Malaria Institute, Weary Dunlop Drive, Enoggera, QLD 4051, Australia; 4 Malaria Clinical Trials Alliance, INDEPTH-Network, Box 213, Kanda, Accra, Ghana

**Keywords:** Microscopy, Species, Morphology, Misclassification, Training

## Abstract

**Background:**

Malaria diagnosis is largely dependent on the demonstration of parasites in stained blood films by conventional microscopy. Accurate identification of the infecting *Plasmodium* species relies on detailed examination of parasite morphological characteristics, such as size, shape, pigment granules, besides the size and shape of the parasitized red blood cells and presence of cell inclusions. This work explores misclassifications of four *Plasmodium* species by conventional microscopy relative to the proficiency of microscopists and morphological characteristics of the parasites on Giemsa-stained blood films.

**Case description:**

Ten-day malaria microscopy remedial courses on parasite detection, species identification and parasite counting were conducted for public health and research laboratory personnel. Proficiency in species identification was assessed at the start (pre) and the end (post) of each course using known blood films of *Plasmodium falciparum, Plasmodium malariae, Plasmodium ovale* and *Plasmodium vivax* infections with densities ranging from 1,000 to 30,000 parasites/μL. Outcomes were categorized as false negative, positive without speciation, *P. falciparum, P. malariae, P. ovale, P. vivax* and mixed infections.

**Discussion and evaluation:**

Reported findings are based on 1,878 P*. falciparum*, 483 *P. malariae,* 581 *P. ovale* and 438 *P. vivax* cumulative results collated from 2008 to 2010 remedial courses. Pre-training false negative and positive misclassifications without speciation were significantly lower on *P. falciparum* infections compared to non-falciparum infections (p < 0.0001). Post-training misclassifications decreased significantly compared to pre- training misclassifications which in turn led to significant improvements in the identification of the four species. However, *P. falciparum* infections were highly misclassified as mixed infections, *P. ovale* misclassified as *P. vivax* and *P. vivax* similarly misclassified as *P. ovale* (p < 0.05).

**Conclusion:**

These findings suggest that the misclassification of malaria species could be a common occurrence especially where non-falciparum infections are involved due to lack of requisite skills in microscopic diagnosis and variations in morphological characteristics within and between *Plasmodium* species. Remedial training might improve reliability of conventional light microscopy with respect to differentiation of *Plasmodium* infections.

## Background

Malaria diagnosis in most settings is largely dependent on the demonstration of parasites in stained blood films by conventional microscopy. This enables parasite detection, identification of *Plasmodium* species and estimation of parasite densities. Accurate identification of the infecting *Plasmodium* species relies on detailed examination of parasite morphological characteristics such as size, shape, pigmentation, besides the size and shape of the parasitized red blood cells and the inclusions therein [1,2]. Medical text books, reference literature and atlases that describe morphological features of *Plasmodium* species already exist to aid microscopic diagnosis of malaria infections [1].

*Plasmodium* species traditionally associated with human infections are *Plasmodium falciparum*, *Plasmodium vivax, Plasmodium malariae* and *Plasmodium ovale*. However, *Plasmodium knowlesi* associated with long tailed macaque monkeys has of late crossed over to humans and is now considered by some to be the fifth human malaria [3,4]. Since *Plasmodium* species differ with respect to their biology, clinical symptoms, and treatment requirements [5,6]; accurate identification of *Plasmodium* infections is of critical importance with regard to formulation and deployment of effective intervention strategies in endemic regions [6].

*Plasmodium falciparu*m is the most prevalent malaria infection in Africa and accounts for the largest proportion of clinical cases [7,8]. *Plasmodium malariae* infections are frequently found in sympatry with *P. falciparu*m infections [7,9-11], but cases go undetected unless molecular methods are used for diagnosis [10]. The burden of *P. ovale* is thought to be highest in sub-Saharan Africa. However, its prevalence is deemed to be lower compared to *P. malariae*[11,12]. The near absence of Duffy positive phenotype in the local populations seems to exclude transmission of *P. vivax* in sub-Saharan Africa [13,14] except in countries such as Djibouti, Eritrea, Ethiopia, Somalia, and Sudan [15].

This work explores misclassification of four *Plasmodium* species by conventional microscopy relative to the proficiency of microscopists and morphological characteristics of the parasites on Giemsa stained blood films.

## Case description

### Methods

#### Remedial microscopy courses

Ten-day remedial malaria microscopy courses were conducted by Kenya Medical Research Institute/US Army Medical Research Unit-Kenya (KEMRI/USAMRU-K), Malaria Diagnostics Centre (MDC) and Kintampo Health Research Center (KHRC), Ghana from 2008 to 2010 as part of a multilateral capacity building strategy for laboratory personnel in public health and research institutions. Training sessions had both theory-based and practice-based components on parasite detection (presence or absence), morphological characterization of *Plasmodium* species, and parasite counting techniques. The practice-based component focused on examination of reference malaria slide sets under the guidance of expert microscopists.

### Malaria slide sets

Slide sets used for training and proficiency assessments consisted of *P. falciparum*, *P. malariae*, *P. ovale* and *P. vivax* infections with parasite densities ranging from 1,000 – 30,000 parasites/μL. Each slide had a thick and thin film stained using buffered 3% Giemsa solution (Sigma-Aldrich GS1L-1 L) for 60 minutes and integrity of the films maintained with microscope cover-glasses (Fisher Scientific 12-543-D) and DPX mountant (Polysciences 13512). Parasitological validation was conducted via multiple independent examinations of coded sets by expert microscopists in addition to *Plasmodium* genus and species-specific PCR assays.

### Proficiency assessments

Assessments in species identification were by way of pre and post-training examination of 20 slides sets with the above mentioned species distributed equally between *P. falciparum* and non-falciparum infections. Pre and post-training test sets were coded differently and participants allowed five minutes to examine each slide and report their findings. Results were evaluated, feedback provided, and hands-on revision exercises conducted under the guidance of expert microscopists.

### Data analysis

1,878 *P. falciparum*, 483 *P. malariae,* 581 *P. ovale* and 438 *P. vivax* cumulative results were collated and outcomes per species categorized as false negative (FN), positive without speciation (PS), *P. falciparum*, *P. malariae P. ovale*, *P. vivax* and mixed infections (MX). Findings per category were converted to proportions and differences within and between *Plasmodium* infections analysed using Marascuilo Chi square test for multiple proportions with *post hoc* comparisons. Significant differences within species were *X*^2^ values ≥ 12.59, df 6, p < 0.05 and between species *X*^2^ values ≥ 7.82, df 3, p < 0.05 [16].

## Results

### Misclassifications on *P. falciparum* infections

**Figure 1:**
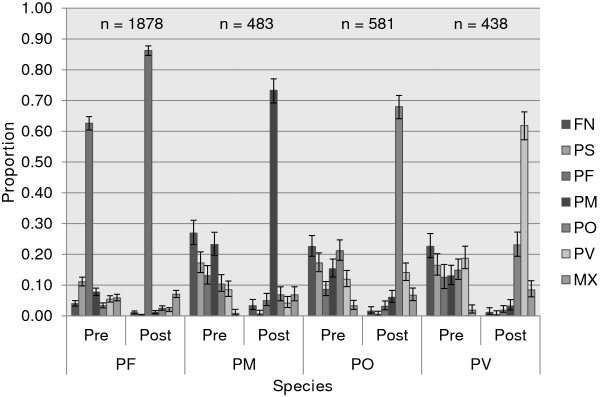
**Cumulative distribution of outcomes across *Plasmodium *species.** Note: 95% CIs constructed using Wilson’s procedure without continuity correction.

Pre-training misclassification of *P. falciparum* infections as PS was significantly higher compared to misclassification as FN, misclassification as *P. ovale,* misclassification as *P. vivax* in addition to misclassification as MX (p < 0.0001). Misclassification as *P. malariae* was significantly higher compared to misclassification as FN together with misclassification as *P. ovale* (p < 0.005) while misclassification as MX was significantly higher compared to misclassification as *P. ovale* (p = 0.029)*.* There were significant reductions in post-training misclassifications except for residual misclassification as *P. ovale* which was marginally lower and residual misclassification as MX which became marginally higher compared to pre-training results (p > 0.05). Residual misclassification as PS was significantly lower compared to residual misclassification as *P. ovale*, residual misclassification as *P. vivax* besides residual misclassification as MX (p < 0.005). Reductions post-training misclassifications significantly improved accurate identification of *P. falciparum* infections compared to pre-training results (Figure 1 & Additional files 1, 2 , 3).

### Misclassifications on *P. malariae* infections

Pre-training misclassification of *P. malariae* infections as FN was significantly higher compared to all the other misclassifications (p < 0.05), misclassification as MX significantly lower compared to the other remaining misclassifications (p < 0.0001) while misclassification as PS was significantly higher compared to misclassification as *P. vivax* (p = 0.012). There were significant reductions in post-training misclassifications except for residual misclassification as *P. ovale* which was marginally lower along with residual misclassification as MX which became significantly higher compared to pre-training results (p < 0.0001). Residual misclassification as PS was significantly lower compared to other residual misclassifications (p < 0.05) except residual misclassification as FN. Reductions in post-training misclassifications resulted in a significant improvement in accurate identification of *P. malariae* infections (Figure 1 & Additional files 1, 2, 3).

### Misclassifications on *P. ovale* infections

Pre-training misclassification of *P. ovale* infections as MX was significantly lower compared to all the other misclassifications (p < 0.05), misclassification as FN significantly higher compared to misclassification as *P. falciparum* together with misclassification *as P. vivax* (p < 0.005) whereas misclassification as PS was higher than misclassification as *P. falciparum* (p = 0.004). There were significant reductions in post-training misclassifications except for residual misclassification as *P. vivax* which became marginally higher and residual misclassification as MX which became significantly higher compared to pre-training results. Notably, residual misclassification of *P. ovale* infections as *P. vivax* became the most common misclassification compared to other residual misclassifications (p < 0.05). This was followed by misclassification as *P. malariae* which became significantly higher than residual misclassification as FN besides to residual misclassification as PS (p < 0.05). The observed reductions in some of the post-training misclassifications contributed to a significant improvement in accurate identification of *P. ovale* infections (Figure 1 & Additional files 1, 2, 3).

### Misclassifications on *P. vivax* infections

Pre-training misclassification of *P. vivax* infections as MX was significantly lower compared to all the other misclassifications (p < 0.0001) whereas misclassification as FN was significantly higher compared to misclassification as *P. falciparum* together with misclassification as *P. malariae* (p < 0.05). There were significant reductions in post-training misclassifications except for residual misclassifications as *P. ovale* along with residual misclassification as MX which became significantly higher compared to pre-training results. Post-training misclassification as *P. ovale* was the most common residual error (p < 0.0001) followed with residual MX when compared to the rest of the residual misclassifications (p < 0.005) except residual misclassification as *P. malariae*. The reductions in post-training misclassifications resulted in a significant improvement in accurate identification of *P. vivax* infections (Figure 1 & Additional files 1, 2, 3).

### Misclassifications between *Plasmodium* infections

Pre-training misclassification of *P. falciparum* infections as FN along with misclassification as PS were significantly lower compared to similar misclassifications on non-falciparum infections (p < 0.05). Misclassification of *P. falciparum* infections as *P. malariae* was equally lower compared to similar misclassification on *P. ovale* and *P. vivax* infections (p < 0.05) and misclassification of *P. falciparum* infections as *P. ovale* significantly lower compared to same misclassification on *P. malariae* and *P. vivax* infections (p < 0.001). Misclassification of *P. falciparum* infections as *P. vivax* was significantly lower compared to equivalent misclassification on *P. ovale* infections (p < 0.001). Misclassification of *P. falciparum* infections as MX was significantly higher compared to similar misclassification of *P. malariae*, *P. ovale* and *P. vivax* infections (p < 0.05). Residual post-training misclassification of *P. falciparum* infections as *P. malariae* was significantly lower compared to equivalent misclassification on *P. ovale* infections (p < 0.001). Misclassification of *P. ovale* infections as *P. vivax* was significantly higher compared to similar misclassifications on *P. falciparum* and *P. malariae* infections respectively (p < 0.001) and misclassification of *P. vivax* infections as *P. ovale* equally higher compared to equivalent misclassification on *P. falciparum* and *P. malariae* infections (p < 0.001) (Figure 1 & Additional file 4).

## Discussion and evaluation

Publications by different groups have shown that laboratory personnel involved in microscopic diagnosis of malaria experience difficulties in differentiating *Plasmodium* infections [17-19]. Those observations are consistent with results presented here with respect to inaccuracies associated with microscopic identification of non-falciparum infections. Higher pre-training false negative and positive results without speciation on non-falciparum infections suggest most of the participants were not familiar with non-falciparum infections and, therefore, opted to report such infections as negative or positive without indicating the appropriate species. Familiarity with non-falciparum during post-training assessments reduced misclassifications within and between species considerably compared to pre-training results.

Lack of significant trends in pre-training misclassification of the four *Plasmodium* infections based on species outcomes were most probably a result of guess work. Nonetheless, significant trends in misclassifications emerged in post-training assessments despite there being significant reductions in species identification errors. These shifts were majorly misclassification of *P. falciparum* infections as mixed infections, misclassification of *P. ovale* infections as *P. vivax* and misclassification of *P. vivax* infections as *P. ovale*.

Misclassification of *P. falciparum* infections as mixed infections can be attributed to morphological variations in parasites forms. Whereas early trophozoites stages are smaller in size and have tiny nucleus and delicate cytoplasm, mature stages are relatively bigger in size, more compact and pigmented. This might have led some participants to believe non- falciparum species were also present on some of the blood films. Misclassification of *P. ovale* infections as *P. vivax* and *vice versa* might have been due to similarities in morphological characteristics such as cell inclusions i.e. James’ and Schüffner’s clefts, host cell enlargement and rounded gametocytes. These overlaps in morphological characteristics make differentiation of the two species extremely challenging even for experienced microscopists. A key feature commonly used in differentiating these two species is the presence of distinct amoeboid or fragmented forms and marked enlargement of the host cell in *P. vivax* infections.

Other than possession of requisite skills in morphological identification, thin films are equally important in enhancing the identification of *Plasmodium* species. Most of the pre-training misclassifications were as a result of thick film examination. While thick films are useful in concentrating parasites for easy identification; the presence of multiple layers of red blood cells which are subsequently de-haemoglobinized during staining distorts essential morphological features needed for species differentiation. Unlike thick films, single layers of red blood cells at the tail of thin films and fixation in alcohol before staining helps in preserving species specific diagnostic details such as red blood cell shape and size, parasite morphology, pigment color and presence of cell inclusions which would otherwise be lost during thick film staining. Therefore, thin films are more reliable in species differentiation compared to thick films. Ideally, thick films should be used for presumptive identification of parasites and estimation of parasite counts whereas thin films should be used for definitive identification of species.

Post-training results did show significant reductions in misclassifications despite the residual errors reported. Occurrence of residual errors can be attributed to variations in morphological characteristics in various stages of parasite development within species and overlap in morphological characteristics between species. It is also important to note that proficiency in microscopic diagnosis of malaria is built over time, based on frequent exposure to different *Plasmodiu*m infections and their respective multiple morphological characteristics.

## Conclusion

Conventional light microscopy despite being the “gold standard” for malaria diagnosis is wrought with many challenges. These findings suggest misclassification of *Plasmodium* infections may be a common occurrence where non-falciparum species are involved. This can be attributed to lack of requisite skills in morphological characterization on the part of microscopists as well as morphological variations in parasite characteristics within and between species. Improving the skill sets of malaria microscopists through remedial training may reduce species misclassification errors associated with microscopy and ensure reliability of patient care and research results. Independent confirmation of species by expert microscopy in addition to molecular analysis of specimens in both patient care setting and research studies is highly recommended.

## Abbreviations

FN: False negative; PS: Positive without speciation; PF: *Plasmodium falciparum*; PM: *Plasmodium malariae*; PO: *Plasmodium ovale*; PV: *Plasmodium vivax*; MX: Mixed infections.

## Competing interests

All authors declared that they have no competing interest.

## Authors’ contributions

PO designed the course, taught the courses, analyzed the data and drafted the manuscript. BO designed the course, facilitated financial and logistical support for courses conducted in Kenya and Ghana and assisted in drafting the manuscript. MA and JSO were the senior instructors of the courses. KL critiqued training methodology and results. CA designed the course, was responsible for logistics and taught the courses. DO was responsible for logistics and taught the courses. SO and FB facilitated financial and logistical support for courses conducted in Ghana. EW and JJ facilitated financial and logistical support for courses conducted in Kenya and assisted in drafting the manuscript. All authors read and approved the final manuscript.

## Supplementary Material

Additional file 1 **FN misclassifications within Plasmodium infections.** Note: Values represent differences between comparisons together with the corresponding X2 statistic, X indicates redundant comparisons while † indicates significant differences. Click here for file

Additional file 2 **PS misclassifications within Plasmodium infections.** Note: Values represent differences between comparisons together with the corresponding X2 statistic, X indicates redundant comparisons while † indicates significant differences. Click here for file

Additional file 3 **Species misclassifications within Plasmodium infections.** Note: Values represent differences between comparisons together with the corresponding X2 statistic, X indicates redundant comparisons while † indicates significant differences. Click here for file

Additional file 4 **Misclassifications between Plasmodium infections.** Note: Values represent differences between comparisons together with the corresponding X2 statistic, X indicates redundant comparisons while † indicates significant differences. Click here for file
