# Supplementary material for: Misclassification of Plasmodium infections by conventional microscopy and the impact of remedial training on the proficiency of laboratory technicians in species identification
Source: Malar J. 2013 Mar 27;12:113. doi: 10.1186/1475-2875-12-113 (PMC3626703; doi:10.1186/1475-2875-12-113)
Supplement: Additional file 1 — FN misclassifications within Plasmodium infections. Note: Values represent differences between comparisons together with the corresponding X2 statistic, X indicates redundant comparisons while † indicates significant differences. [file 1475-2875-12-113-S1.docx]

|  |  | **FN vs. PS** | **FN vs. PF** | **FN vs. PM** | **FN vs. PO** | **FN vs. PV** | **FN vs. MX** |
| --- | --- | --- | --- | --- | --- | --- | --- |
| *P. falciparum* | Pre | -0.071, 68.84† | X | -0.037, 23.81† | 0.007, 1.28 | -0.014, 4.33 | -0.019, 6.98 |
|  | Post | 0.009, 10.77 | X | 0.000, 0.00 | -0.014, 10.45 | -0.009, 4.65 | 0.060, 88.02† |
| *P. malariae* | Pre | 0.097, 13.49† | 0.139, 29.97† | X | 0.166, 45.77† | 0.184, 50.77† | 0.261, 160.40† |
|  | Post | 0.027, 9.16 | -0.017, 1.67 | X | -0.035, 6.25 | -0.008, 0.46 | -0.035, 6.25 |
| *P. ovale* | Pre | 0.053, 5.22 | 0.139, 44.58† | 0.072, 9.98 | X | 0.107, 23.69† | 0.193, 104.67† |
|  | Post | 0.010, 3.04 | -0.016, 3.08 | -0.045, 16.19† | X | -.126, 67.21† | -0.052, 19.89† |
| *P. vivax* | Pre | 0.062, 5.33 | 0.101, 15.52† | 0.096, 13.98† | 0.078, 8.76 | X | 0.208, 98.03† |
|  | Post | -0.007, 1.30 | -0.007, 0.70 | -0.021, 4.38 | -0.219, 111.50† | X | -0.073, 26.38† |
