# Supplementary material for: Misclassification of Plasmodium infections by conventional microscopy and the impact of remedial training on the proficiency of laboratory technicians in species identification
Source: Malar J. 2013 Mar 27;12:113. doi: 10.1186/1475-2875-12-113 (PMC3626703; doi:10.1186/1475-2875-12-113)
Supplement: Additional file 2 — PS misclassifications within Plasmodium infections. Note: Values represent differences between comparisons together with the corresponding X2 statistic, X indicates redundant comparisons while † indicates significant differences. [file 1475-2875-12-113-S2.docx]

|  |  | **PS vs. PF** | **PS vs. PM** | **PS vs. PO** | **PS vs. PV** | **PS vs. MX** |
| --- | --- | --- | --- | --- | --- | --- |
| *P. falciparum* | Pre | X | 0.034, 12.45 | 0.078, 87.03† | 0.056, 39.93† | 0.052, 33.29† |
|  | Post | X | -0.009, 10.77 | -0.022, 36.10† | -0.017, 26.06† | -0.068, 129.30† |
| *P. malariae* | Pre | 0.041, 3.24 | X | 0.068, 9.59 | 0.087, 16.60† | 0.164, 85.84† |
|  | Post | -0.044, 17.10† | X | -0.62, 26.69† | -0.035, 13.05† | -0.062, 26.69† |
| *P. ovale* | Pre | 0.086, 19.46† | 0.019, 0.77 | X | 0.053, 6.69 | 0.139, 64.85† |
|  | Post | -0.026, 11.01 | -0.055, 28.54† | X | -0.136, 85.01† | -0.062, 32.92† |
| *P. vivax* | Pre | 0.039, 2.67 | 0.034, 2.05 | 0.016, 0.42 | X | 0.146, 60.22† |
|  | Post | -0.014, 3.66 | -0.027, 9.26 | -0.226, 122.97† | X | -0.080, 34.16† |
