# Supplementary material for: Misclassification of Plasmodium infections by conventional microscopy and the impact of remedial training on the proficiency of laboratory technicians in species identification
Source: Malar J. 2013 Mar 27;12:113. doi: 10.1186/1475-2875-12-113 (PMC3626703; doi:10.1186/1475-2875-12-113)
Supplement: Additional file 3 — Species misclassifications within Plasmodium infections. Note: Values represent differences between comparisons together with the corresponding X2 statistic, X indicates redundant comparisons while † indicates significant differences. [file 1475-2875-12-113-S3.docx]

|  | **P. falciparum** | | **P. malariae** | | **P. ovale** | | **P. vivax** | |
| --- | --- | --- | --- | --- | --- | --- | --- | --- |
|  | **Pre** | **Post** | **Pre** | **Post** | **Pre** | **Post** | **Pre** | **Post** |
| PF vs. PM | X | X | X | X | -0.067, 12.56 | -0.029, 5.74 | -0.005, 0.04 | -0.014, 1.68 |
| Pf vs. PO | X | X | 0.027, 1.70 | -0.019, 1.51 | X | X | -0.023, 0.97 | -0.212, 101,08† |
| PF vs. PV | X | X | 0.046, 5.24 | 0.008, 0.38 | -0.033, 3.39 | -0.110, 46.61† | X | X |
| PF vs. MX | X | X | 0.122, 59.25† | -0.019, 1.51 | 0.053, 15.00† | -0.036, 8.19 | 0.107, 39.48† | -0.066, 20.15† |
| PM vs. PO | 0.044, 35.55† | -0.014, 10.45 | X | X | X | X | -0.018, 0.61 | -0.199, 82.94† |
| PM vs. PV | 0.023, 8.03 | -0.009, 4.65 | X | X | -0.034, 2.94 | -0.081, 21.38† | X | X |
| PM vs. MX | 0.019, 5.16 | -0.060, 88.02† | X | X | 0.121, 52.27† | -0.007, 0.23 | 0.112, 41.80† | -0.053, 11.15 |
| PO vs. PV | -0.021, 10.23 | 0.005, 1.25 | 0.019, 0.98 | 0.027, 3.39 | X | X | X | X |
| PO vs. MX | -0.026, 14.09† | -0.046, 44.13† | 0.095, 43.37† | 0.000, 0.00 | X | X | 0.130, 51.40† | 0.146, 36.71† |
| PV vs. MX | -0.004, 0.32 | -0.051, 58.32† | 0.077, 33.00† | -0.027, 3.39 | 0.086, 31.57† | 0.074, 17.31† | X | X |
