# Supplementary material for: Misclassification of Plasmodium infections by conventional microscopy and the impact of remedial training on the proficiency of laboratory technicians in species identification
Source: Malar J. 2013 Mar 27;12:113. doi: 10.1186/1475-2875-12-113 (PMC3626703; doi:10.1186/1475-2875-12-113)
Supplement: Additional file 4 — Misclassifications between Plasmodium infections. Note: Values represent differences between comparisons together with the corresponding X2 statistic, X indicates redundant comparisons while † indicates significant differences. [file 1475-2875-12-113-S4.docx]

|  |  | **FN** | **PS** | **PF** | **PM** | **PO** | **PV** | **MX** |
| --- | --- | --- | --- | --- | --- | --- | --- | --- |
| PF vs. PM | Pre | -0.229, 122.85† | -0.061,10.75† | X | X | -0.017,104.95† | -0.031, 4.97 | 0.050, 54.56† |
|  | Post | 0.023, 6.85 | -0.004, 1.20 | X | X | -0.044, 13.29† | -0.022, 5.36 | 0.002, 0.023 |
| PF vs. PO | Pre | -0.186, 107.24† | -0.061, 12.65† | X | -0.076, 22.10† | X | -0.064, 20.02† | 0.026, 7.99† |
|  | Post | 0.005, 0.71 | -0.003, 0.92 | X | -0.050, 23.87† | X | -0.122, 68.04† | 0.003, 0.07 |
| PF vs. PV | Pre | -0.186, 82.49† | -0.054, 7.86† | X | -0.053, 9.45† | -0.115, 43.58† | X | 0.040, 23.11† |
|  | Post | -0.001, 0.01 | -0.002, 0.52 | X | -0.021, 5.96 | -0.206,101.67† | X | -0.014, 0.95 |
| PM vs. PO | Pre | 0.044, 2.70 | 0.000, 0.00 | 0.044, 5.32 | X | X | -0.034, 3.37 | -0.024, 23.11† |
|  | Post | 0.017, 3.22 | 0.001, 0.051 | 0.019, 2.34 | X | X | -0.100, 34.20† | 0.001, 0.01 |
| PM vs. PV | Pre | 0.043, 2.31 | 0.001, 0.092 | 0.005, 0.049 | X | -0.045, 4.19 | X | -0.010, 1.72 |
|  | Post | 0.021, 4.94 | 0.002, 0.12 | 0.031, 7.12 | X | -0.162, 49.05† | X | -0.016, 0.85 |
| PO vs. PV | Pre | -0.001, 0.00 | 0.001, 0.11 | -0.040, 4.04 | 0.023, 1.10 | X | X | 0.0144, 2.19 |
|  | Post | 0.004, 0.31 | 0.001, 0.019 | 0.013, 1.75 | 0.028, 4.76 | X | X | -0.017, 1.05 |
